# Publisher Correction: Multidimensional natal isotopic niches reflect migratory patterns in birds

**DOI:** 10.1038/s41598-021-01429-6

**Published:** 2021-11-04

**Authors:** A. Franzoi, S. Larsen, P. Franceschi, K. A. Hobson, P. Pedrini, F. Camin, L. Bontempo

**Affiliations:** 1 Vertebrate Zoology Department, MUSE – Science Museum, Corso del Lavoro e della Scienza 3, 38122 Trento, TN Italy; 2 Research and Innovation Centre, Fondazione Edmund Mach, via E. Mach 1, 38010 San Michele all’Adige, Italy; 3 Department of Earth and Environmental Sciences, Università degli Studi di Pavia, Via Ferrata 1, 27100 Pavia, Italy; 4 Environment and Climate Change Canada, Innovation Blvd., Saskatoon, SK S7N 3H5 Canada; 5 Center Agriculture Food Environment (C3A), University of Trento, Via Mach 1, 38010 San Michele all’Adige, TN Italy; 6 Present Address: Vienna International Centre, International Atomic Energy Agency, PO Box 100, 1400 Vienna, Austria

Correction to: *Scientific Reports* https://doi.org/10.1038/s41598-021-00373-9, published online 21 October 2021

The original version of this Article contained an error in Figure 1 where the labels for the species and individuals were missing from the distributions. The original Figure 1 and accompanying legend appear below.

**Figure 1:**
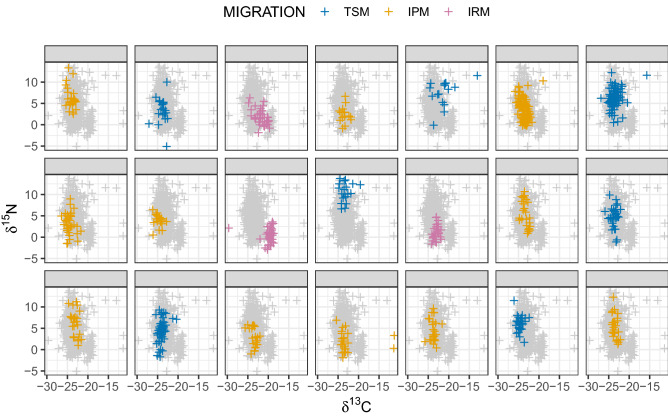
Distribution of each species and individuals over the *δ*^13^C-*δ*^15^N isotope space defining the dietary niche. Grey crosses in the background show the overall space occupied by the species (acronyms provided in Table S1). *TSM* Trans-Saharan migrants, *IPM* Intra-Palearctic migrants, *IRM* Irruptive migrants.

The original Article has been corrected.

