# Supplementary material for: Multidimensional natal isotopic niches reflect migratory patterns in birds
Source: Sci Rep. 2021 Oct 21;11:20800. doi: 10.1038/s41598-021-00373-9 (PMC8531022; doi:10.1038/s41598-021-00373-9)
Supplement: Supplementary file 1 — Supplementary Information. [file 41598_2021_373_MOESM1_ESM.docx]

**Multidimensional natal isotopic niches reflect migratory patterns in birds**

Franzoi A^1,2^., Larsen S.*^3^, Franceschi P.^3^, Hobson K.A.^4^., Pedrini P. ^1^ Camin F.^3^ & Bontempo L.^3^

**Supplementary Material**

Table S1 – List of bird species included in the analyses. Latin and English names are given, with acronyms used in the plots.

**
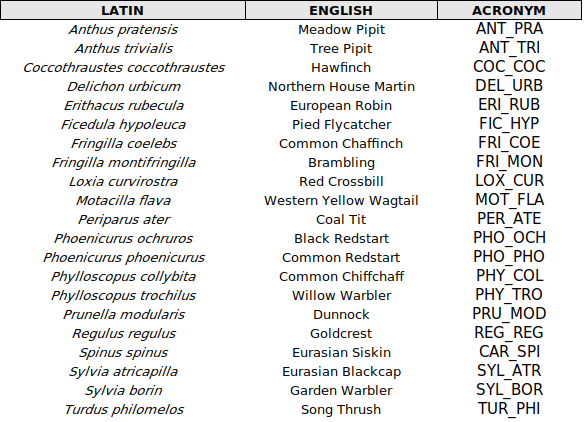
**


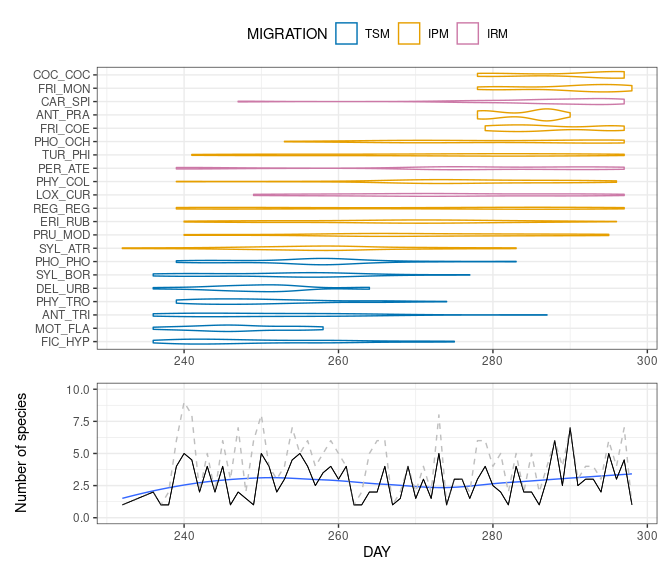


**Fig. S1** - Migratory phenology of the 21 species during the autumn migration over the Italian Alps. Violin plots were built combining four years of sampling (2010-2013).

Lower panel shows the median (across years; black line) and maximum (grey dashed line) number of species captured each day. A LOESS smooth of the median is also shown. TSM=Trans-Saharan migrants; IPM=Intra-Palearctic migrants; IRM=Irruptive migrants


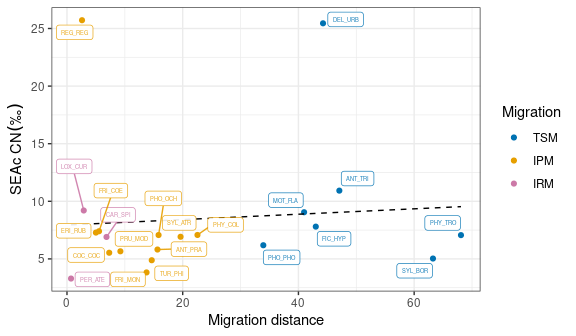


**Fig. S2**- Relationship between species dietary NB and migration distance, defined as the difference between the mean latitude of the breeding and wintering ranges. Species are labelled. TSM=Trans-Saharan migrants; IPM=Intra-Palearctic migrants; IRM=Irruptive migrants


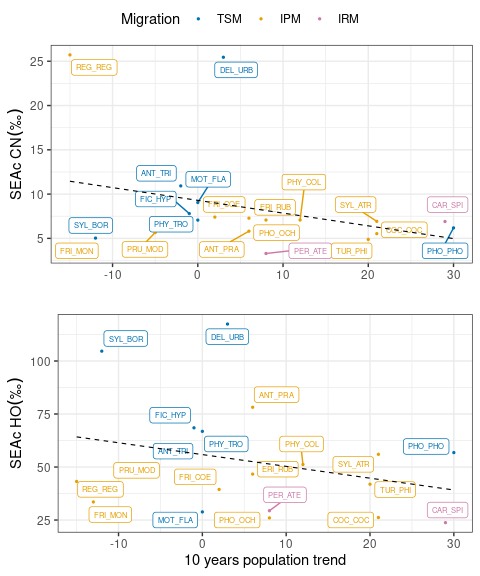


**Fig. S3** - Relationship between isotopic dietary and origin breeding niche breadth and population trends as reported in the Pan European Common Bird Monitoring Scheme. Dashed line indicates non-significant trends. Species are labelled. TSM=Trans-Saharan migrants; IPM=Intra-Palearctic migrants; IRM=Irruptive migrants


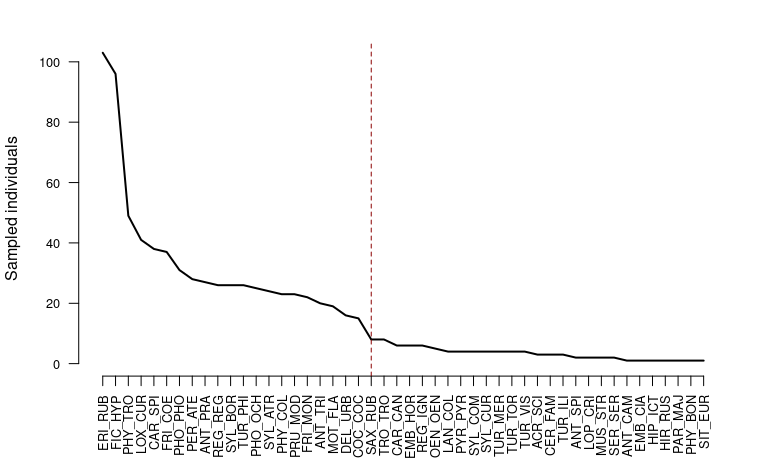


**Fig. S4 –** Number of sampled individuals for each species. Dashed vertical line shows the chosen cut-off level of 15 individuals for the inclusion of species in the analyses.


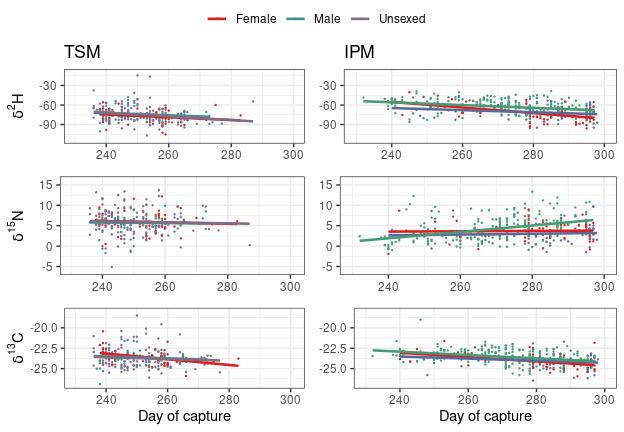


**Fig. S5**- Temporal trends in *δ*^2^H, *δ*^15^N and *δ*^13^C values for the TSM, IPM and IRM groups, coloured by sex. Male and female displayed similar patterns.


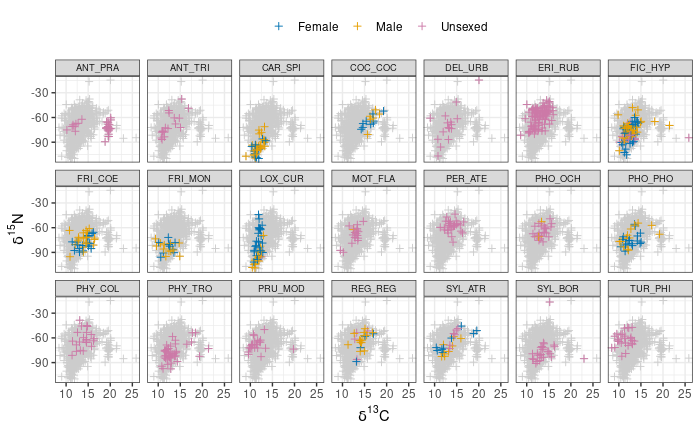


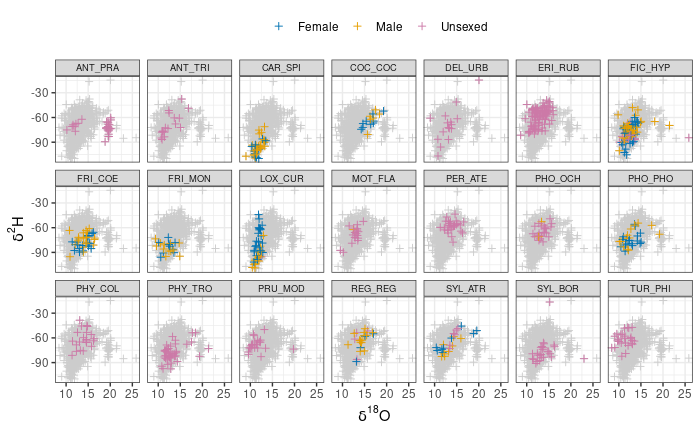


**Fig. S6** - Distribution of each species and individuals over the *δ*^13^C-*δ*^15^N and *δ*^2^H-*δ*^18^O isotope space, coloured by sex
